# Mechanical Stretch Induces Apoptosis Regulator TRB3 in Cultured Cardiomyocytes and Volume-Overloaded Heart

**DOI:** 10.1371/journal.pone.0123235

**Published:** 2015-04-21

**Authors:** Wen-Pin Cheng, Bao-Wei Wang, Huey-Ming Lo, Kou-Gi Shyu

**Affiliations:** 1 Department of Medical Education and Research, Shin Kong Wu Ho-Su Memorial Hospital, Taipei, Taiwan; 2 Division of Cardiology, Shin Kong Wu Ho-Su Memorial Hospital, Taipei, Taiwan; 3 School of Medicine, Fu-Jen Catholic University, New Taipei City, Taiwan; 4 Graduate Institute of Clinical Medicine, College of Medicine, Taipei Medical University, Taipei, Taiwan; Faculty of Medicine & Health Sciences, UNITED ARAB EMIRATES

## Abstract

The expression of TRB3 (tribbles 3), an apoptosis regulated gene, increases during endoplasmic reticulum (ER) stress. How mechanical stress affects the regulation of TRB3 in cardiomyocytes during apoptosis is not fully understood. An in vivo model of aorta-caval shunt in adult rats demonstrated the increased TRB3 protein expression in the myocardium. The tumor necrosis factor-alpha (TNF-α) antagonist etanercept reversed the TRB3 protein expression and cardiomyocyte apoptosis induced by AV shunt. An in vitro model of cyclic stretch in neonatal rats was also used to investigate TRB3 expression. We hypothesized that cardiomyocyte apoptosis induced by cyclic stretch is TRB3 dependent. Neonatal rat cardiomyocytes grown on a flexible membrane base were stretched by vacuum to 20% of maximum elongation, at 60 cycles/min. Cyclic stretch significantly increased TRB3 protein and mRNA expression. Addition of c-jun N-terminal kinase (JNK) inhibitor SP600125, TNF-α antibody and etanercept 30 min before stretch reversed the induction of TRB3 protein induced by stretch. Cyclic stretch induced the DNA-binding activity of growth arrest and DNA damaged inducible gene-153 (GADD153) by electrophoretic mobility shift assay. SP600125, JNK siRNA, TNF-α antibody and etanercept abolished the binding activity induced by stretch. TRB3 promoter activity was enhanced by stretch and TRB3-mut plasmid, SP600125, TNF-α antibody and etanercept attenuated TRB3 promoter activity induced by stretch. Exogenous administration of TNF-α recombinant protein to the non-stretched cardiomyocytes increased TRB3 protein expression similar to that seen after stretch. Cyclic stretch induced cardiomyocyte apoptosis is inhibited by TRB3 siRNA and etanercept. The stretch-induced TRB3 is mediated by TNF-α、JNK and GADD153 pathway. These results indicate that TRB3 plays an important role in stretch-induced cardiomyocyte apoptosis.

## Introduction

Cardiovascular disease with cardiac hypertrophy is a leading cause of death in the Western countries. Cardiac hypertrophy is often accompanied by cardiac remodeling characterized by cardiomyocyte loss, interstitial fibrosis and collagen deposition and increases the risk of heart failure [1]. Cardiomyocyte apoptosis is an important factor during the transition from compensatory hypertrophy to heart failure [2]. Cardiomyocyte apoptosis has been reported in a variety of cardiovascular diseases, including ischemia/reperfusion [3], end-stage heart failure, myocardial infarction [4], right ventricular dysplasia and cardiomyopathy [5]. The role of cardiomyocyte apoptosis in the progression of cardiac disease remains controversial. Therefore, the possibility of reducing cardiomyocytes loss by inhibiting apoptosis has potentially important implications in the treatment of heart failure.

Endoplasmic reticulum (ER) stress can induce cardiac cells apoptosis in association with cardiac disease [6]. CCAAT-enhancer-binding protein homologous protein (CHOP) /growth arrest and DNA damage inducible gene 153 (GADD153) is a major molecular component involved in ER stress-induced apoptosis [7]. Although the exact role of GADD153 in the ER stress response is not fully understood, it has been shown that GADD153-mediated apoptosis is through induction of tribbles 3 (TRB3) in a variety type of cells [8].

TRB3, also named neuronal cell death-inducible putative protein kinase, is expressed in the liver, thymus, prostate and heart [9]. TRB3 is an important regulatory protein involved in Akt and MAPK pathway [10, 11]. It is also a novel target of GADD153/ATF4 and the tunicamycin response region in the TRB3 promoter contains amino-acid response elements overlapping the GADD153-binding site [12]. ER stress inducers, such as tunicamycin, thapsigargin, the long chain fatty acid palmitate, and hypoxia all enhance the expression of TRB3. ER stress caused by myocardial infarction in the infarct border zone was associated with TRB3 induction in cardiomyocytes [13]. Besides, knockdown of TRB3 expression attenuates the ER stress-dependent apoptosis [14]. TRB3 is also involved in fibrosis [15], atherosclerosis [16] and insulin resistance [17]. However, the role of TRB3 in cardiovascular disease is still controversial.

Mechanical force overload is able to induce inflammatory mediators and causes ventricular hypertrophy [18]. To determine the molecular pathways involved in the hypertrophic response to mechanical stress, in vitro stretching devices have been developed that enable stretch stress to be applied to cultured cardiomyocytes. Cyclic stretch could induce ER related apoptotic gene GADD153 and cardiomyocyte apoptosis [19]. There was also an evidence to show that TRB3 plays an important role in cardiomyocyte apoptosis upon ER stress [20]. However, there is no conclusive proof on how cyclic stretch affects the TRB3 expression and the relationship between TRB3 and GADD153 on the cardiomyocyte apoptosis. Besides, we used the animal model aorta-caval shunt, a volume-overload model, to investigate the expression of TRB3 in the left ventricular myocardium. Tumor necrosis factor-α (TNF-α) is a major inflammatory cytokine that inducing apoptosis under stress. Etanercept, a TNF-α antagonist, is used to treat chronic inflammatory diseases and could reduce cardiomyocyte apoptosis induced by mechanical trauma [21]. However, the effect of etanercept on TRB3-mediated myocardial apoptosis induced by AV-shunt and mechanical cyclic stretch is still unknown. Therefore, we also used etanercept to inhibit the TRB3 expression and cardiomyocyte apoptosis under AV-shunt and cyclic stretch.

## Materials and Methods

### Ethical statement

The male Wistars rat that purchasing from BioLASCO (Yilan, Taiwan) were feed and housing in auditory, visual, toys, and hideaways enrichment in accordance with the standards of the Committee of Animal Care and Use of Shin Kong Wu Ho-Su Memorial Hospital. All animal study protocols were approved by our Committee of Animal Care and Use of Shin Kong Wu Ho-Su Memorial Hospital (permit number:1021025015) and were carried out in accordance with the Guide for the Care and Use of Laboratory Animals (NIH publication No. 86–23, revised 2011). Animal study was performed after confirming a fully anaesthetized state (.e.g. no response to toe pinching). All efforts were made to ameliorate the welfare and to minimize suffering according to the Institutional Animal Care and Use Committee.

### Cardiomyocytes culture

Cardiomyocytes were obtained from Wistar rats aged 2–3 days old by trypsinization as previously described [19]. Cultured cardiomyocytes thus obtained were > 95% pure as revealed by observation of contractile characteristics with a light microscope and stained with anti-desmin antibody (Dako Cytomation, Glostup, Denmark). Cardiomyocytes were seeded on cultured dish in Ham’s F-10 containing 20% fetal bovine serum. After 3days in culture, cells were transferred to serum-free Dulbecco’s modified Eagle’s medium (DMEM) and subjected to stretch.

### In vitro cyclic stretch on cultured cardiomyocytes

The Flexcell FX-2000 strain unit, which has been characterized and described in detail elsewhere [19]. To determine the roles of c-Jun N-terminal kinase (JNK), p38 MAP kinase or p44 MAP kinase in the expression of stretch-induced TRB3 expression, cardiomyocytes were pretreated with SP600125 (20 μM, Calbiochem, San-Diego, California, USA), SB203580 (3 μM, Calbiochem) or PD98059 (50 μM, Calbiochem) for 30 mins, respectively, followed by stretch. SP600125 is a specific inhibitor of JNK. PD98059 is a specific inhibitor of ERK kinase. SB203580 is a highly specific inhibitor of p38 kinase.

### Reverse transcription (RT) polymerase chain reaction

RT-PCR was performed as previously described [19].

### Real-time Quantitative PCR

The primers used were as follows: TRB3, 5′-d(TCGCTGACCGTGAGAGGAA)-3′ (forward) and 5′-d(GCAAGATGAGCCCGTGCTT)-3′ (reverse); and glyderaldehyde-3-phosphate dehydrogenase (GAPDH), 5′-d(CATCACCATCTTCCAGGAGC) (forward) and 5′-d(GGATGATGTTCTGGGCTGCC)-3′ (reverse). Details of the procedures are further described in the S1 Methods.

### Western blot analysis

A western blot was performed as previously described [19]. Antibodies used for the western blot are described in detail in the S1 Methods.

### Electrophoretic mobility shift assay (EMSA)

GADD153 binding site used was TGGTGCAATCCCC [22]. The GADD153 mutant oligonucleotides sequences were TGGTATAATCCCC. The methodology for the EMSA is further described in detail in the S1 Methods.

### Construction of small interfering RNA (siRNA)

TRB3 siRNA: sense: 5’-P.CAGCCUACCUCCCGCCUCA-3’ and antisense: 5’-P.UGAGGCGGGAG UAGGCUG-3’. For negative control, scramble siRNA for TRB3 was used, sense: 5’-P.UCCACCCGUUCGACCCACC-3’ and antisense: 5’-P.GGUGGGUCGAAC GGUGGA-3’ (Dharmacon). GADD153 siRNA: sense: 5’-P.-GGUAUGAGGAUCUGCAGGAUU-3’; antisense: 5’-P.UCCUGCAGAUCCU CAUACCUU-3’ (Dharmacon). JNK1 siRNA: sense: 5’-P.CGUGGAUUUAUGG UCUGUGdTdT-3’; antisense: 5’-P.CACAGACCAUAAAUCCACCdTdT-3’ (Dharmacon). After overnight incubation, cells were stretched and subjected to analysis by western blot, EMSA, immunohistochemistry and detection of apoptosis. Details of the procedures are further described in the S1 Methods.

### Promoter activity assay

The TRB3 promoter construct containing the GADD153 binding site were transfected into cardiomyocytes using a low pressure-accelerated gene gun (Bioware Technologies, Taipei, Taiwan) as previously described [19].

### Measurement of TNF-α concentration by enzyme-linked immunosorbent assay (ELISA)

Conditioned medium from cardiomyocytes subjected to stretch and those from control (unstretched) cells were collected for TNF-α measurement. The level of TNF-α was measured by a quantitative sandwich enzyme immunoassay technique. The lowest limit of TNF-α ELISA kit is 54 pg/ml.

### Flow cytometric analysis for apoptotic quantitation

Apoptotic cells were quantified as the percentage of cells stain with annexin V. Cardiomyocytes were fixed with 70% ethanol and treated with RNase. Then nuclei were stained with propidium iodide and annexin V (Millipore, Temecula, CA, USA). The DNA content was measured using by a FACSCalibur flow cytometer and Cell Quest software (Becton Dickinson, Franklin Lakes, NJ, USA). For all assays, ten thousand cells were counted.

### Terminal deoxynucleotidyl transferase-mediated dUTP nick-end labeling (TUNEL) assay

DNA fragmentation was determined via terminal deoxynucleotidyl transferase-mediated dUTP nick-end labeling (TUNEL) using the ApopTag peroxidase in situ apoptosis detection kit (Chemicon International, Temecula, CA, USA). The methodology for the TUNEL assay is further described in detail in the S1 Methods.

### Rat model of aorta-caval shunt

On the day of surgery, the Wistar male rats weighing 250 to 300 g were anesthetized with isoflurane (80 mg/kg). The vena cava and aorta were exposed via abdominal midline incision after confirming a fully anaesthetized state (.e.g. no response to toe pinching). In brief, the aorta was punctured at the union of the segment two-thirds caudal to the left renal artery and one-third cephalic to the aortic bifurcation, with an 18 gauge disposable needle held with a plastic syringe. Sham-operated control animals were prepared similar manner, except that the aorta was not punctured. For AV shunt time course study, rats were randomly divided into five groups (1) sham-operated (n = 5), (2) AV shunt 3 days (n = 7), (3) AV shunt 5 days (n = 7), (4) AV shunt 7 days (n = 7), (5) AV shunt 14 days (n = 7). For AV shunt and treatment of etanercept study, rats were randomly divided into four groups (1) sham-operated (n = 5), (2) sham-operated and treatment with etanercept (n = 6), (3) AV shunt 7days (n = 7), (4) AV shunt 7 days and treatment with etanercept (n = 7). The number of rats we used in the in vivo study is fifty eight. Etanercept at 1 mg/kg was given by subcutaneous injection for 7 days after induction of AV shunt. At the end of experiment, rats were euthanized with an overdose of isoflurane. Left ventricular tissue was obtained for Western blot analysis and immunohistochemical staining. We monitored the condition of rats twice per day after surgery.

### Statistical analysis

All results were expressed as mean ± S.E.M. Statistical significance was evaluated with analysis of variance (GraphPad Software Inc., San Diego, CA, USA). The Dunnett’s test was used to compare multiple groups to a single control group. Turkey-Kramer comparison was used for pairwise comparisons between multiple groups after the ANOVA. A value of P<0.05 was considered to denote statistical significance.

## Results

### Cyclic stretch enhances TRB3 protein and mRNA expression in cardiomyocytes

**Fig 1:**
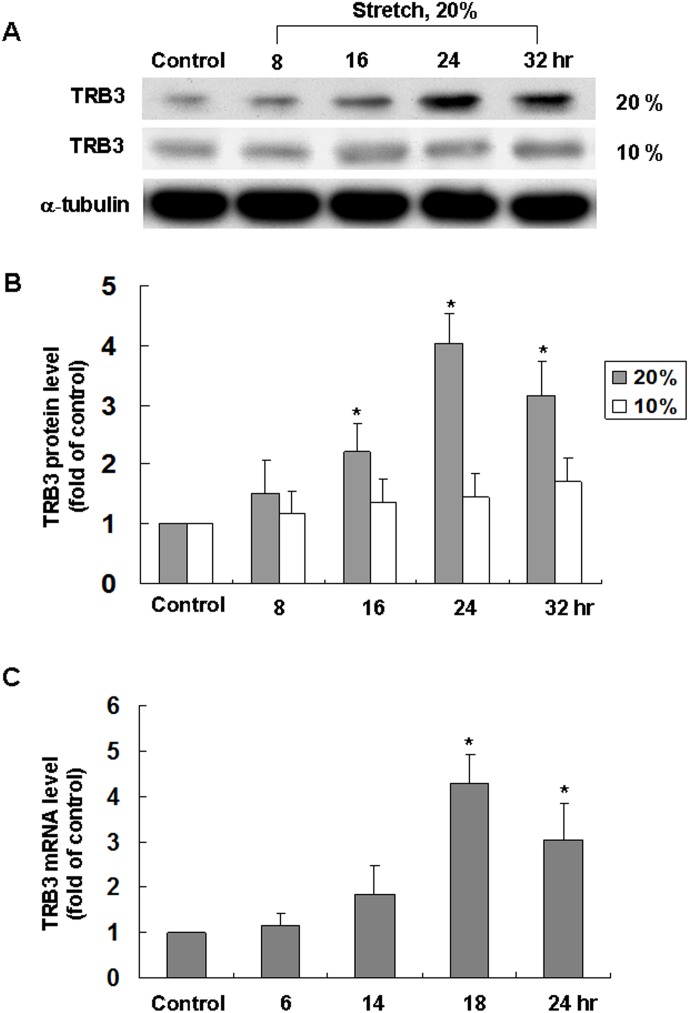
Cyclic stretch induces TRB3 protein expression in cardiomyocytes. (A) Representative Western Blots for TRB3 in cardiomyocytes subjected to cyclic stretch by 10% or 20% for various periods of time. Control group means non-stretched cardiomyocytes and they were collected at 32h. (B) Quantitative analysis of TRB3 protein levels. The values from stretched cardiomyocytes have been normalized to matched α-tubulin measurement and then expressed as a ratio of normalized values to protein in control group (n = 4 per group). *P < 0.05 vs. control. (C) Quantitative analysis of TRB3 mRNA levels in cardiomyocytes subjected to stretch by 20% for various periods of time. The values from stretched cardiomyocytes have been normalized to matched GAPDH measurement and then expressed as a ratio of normalized values to mRNA in control group (n = 4 per group). *P < 0.05 vs. control. Control group means non-stretched cardiomyocytes and they were collected at 24h.

The level of TRB3 protein began to increase as early as 16 h after stretch to 20%, reached a maximum of 4-fold over the control by 24 h, and remained elevated up to 32 h. When cardiomyocytes were stretched at 10% elongation, the level of TRB3 protein was similar to that of control without stretch (Fig 1A and 1B). The real-time PCR showed that TRB3 messages RNA increased significantly after 18 h of stretch at 20% (Fig 1C). These results indicate that cyclic stretch induces TRB3 expression in cardiomyocytes.

### Stretch-induced TRB3 protein expression in cardiomyocytes is mediated by JNK and GADD153

**Fig 2:**
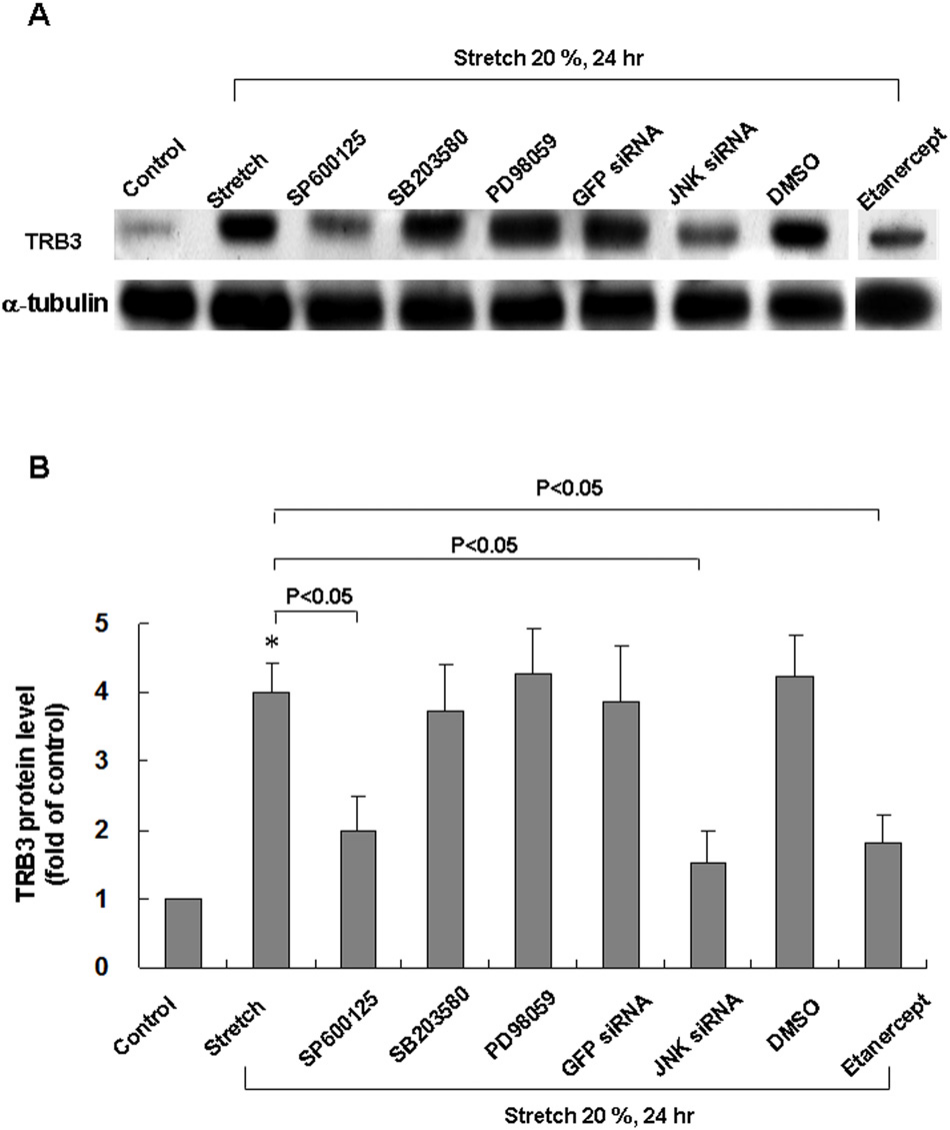
Effects of MAPK inhibitors or etanercept on TRB3 protein expression induced by cyclic stretch in cardiomyocytes. (A) Representative Western Blots for TRB3 protein levels in cardiomyocytes subjected to cyclic stretch in the absence or presence of MAPK inhibitors, siRNA, etanercept (1 μg/ml, purchased from BOC Science, NY, USA) and vehicle (DMSO 0.1%). (B) Quantitative analysis of TRB3 protein levels. The values from stretched cardiomyocytes have been normalized to matched α-tubulin measurement and then expressed as a ratio of normalized values to protein in control group (n = 3 per group). *P<0.05 vs. control.

To investigate the possible signal pathway mediating the stretch-induced TRB3 in cardiomyocytes, the cardiomyocytes were stretched 20% for 24 h in the presence or absence of inhibitors or siRNA. As shown in Fig 2, the stretch-induced increases of TRB3 proteins were significantly blocked after addition of SP600125 30 min before stretch. TRB3 proteins induced by stretch were not affected by the addition of PD98059 and SB203580. To test the specific effect of suppressing JNK MAP kinase pathway on JNK phosphorylation (S1 Fig) and TRB3 expression (Fig 2), JNK siRNA was transfected to cardiomyocytes before cyclic stretch. The siRNA treatment significantly reduced the TRB3 level induced by stretch (Fig 2). The DMSO alone as a vehicle control and control siRNA did not affect the TRB3 expression induced by cyclic stretch. These findings implicate that JNK pathway, but not p42/p44 or p38 MAP kinases, mediates the induction of TRB3 proteins by stretch in cardiomyocytes. Besides, we found that TRB3 protein expression induced by stretch is reversed by etanercept (Fig 2) or GADD153 siRNA (S2A and S2B Fig). GADD153 protein expression induced by stretch was inhibited by the addition of GADD153 siRNA (S2C and S2D Fig). This result indicates TRB3 protein expression induced by stretch is mediated by GADD153. Etanercept could reduce TRB3 protein expression induced by stretch.

### Cyclic stretch enhances GADD153 binding activity and increases TRB3 promoter activity

**Fig 3:**
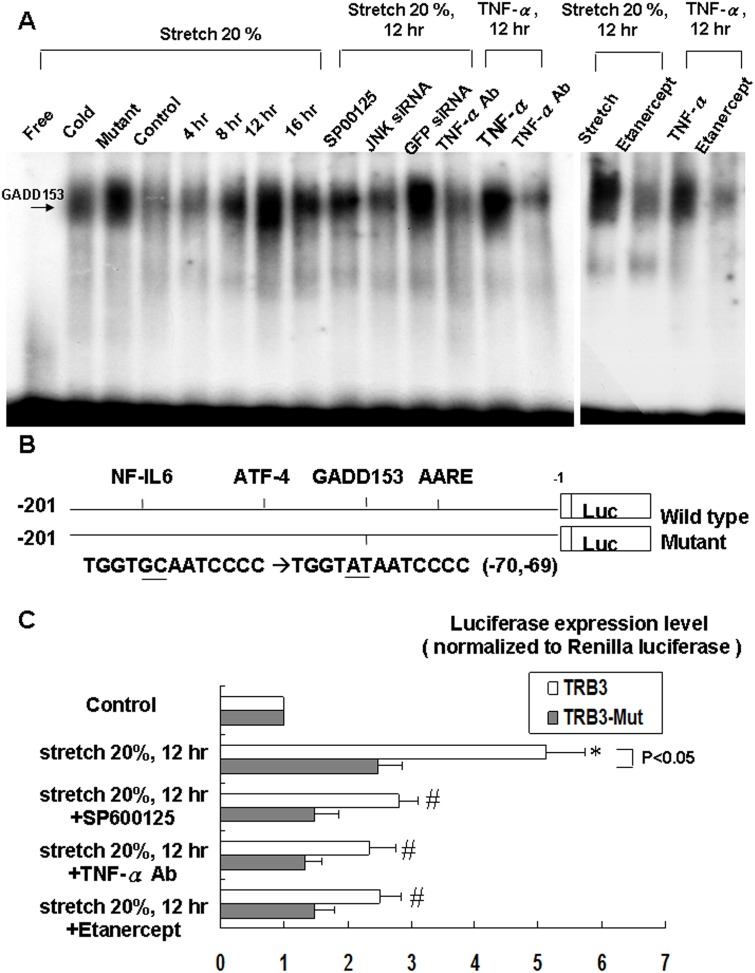
Effects of cyclic stretch on GADD153 binding activity and TRB3 promoter activity in cardiomyocytes. (A) Representative EMSA showing protein binding to GADD153 oligonucleotide in nuclear extracts of cardiomyocytes following cyclic stretch during various times and in the absence or presence of JNK inhibitors or siRNA, TNF-α antibody, etanercept (1 μg/ml) or addition of TNF-α. Arrows indicate the mobility of the complex. Similar results were found in another two independent experiments. Cold oligo means unlabeled GADD153 oligonucleotide. Mutant oligo means an oligonucleotide containing a 2-bp substitution in the GADD153 binding site. The mutant or cold oligo were used for competing. (B) Constructs of TRB3 promoter gene. (C) Quantitative analysis of TRB3 promoter activity. Cardiomyocytes were transiently transfected with pTRB3-Luc by a gene gun. The luciferase activity in cell lysates was measured and was normalized with Renilla activity (n = 3 per group). *P < 0.05 vs. control. ^#^ *P* < 0.05 vs. stretch 12h.

Cyclic stretch significantly began to increase the DNA-protein binding activity of GADD153 in cardiomyocytes at 8 h after stretch and reached a maximum at 12 h and remained elevated for 16 h (Fig 3A). An excess of unlabeled GADD153 oligonucleotide competed with the probe for binding GADD153 protein, whereas an oligonucleotide containing a 2-bp substitution in the GADD153 binding site did not compete for binding. Addition of SP600125, TNF-α antibody (5 μg/ml, purchased from R&D Systems) and etanerept 30 min or JNK siRNA 24h prior to stretch abolished the DNA-protein binding activity induced by stretch. Exogenous TNF-α also induced GADD153 binding activity. These results demonstrate that stretch enhanced GADD153 binding activity is mediated by TNF-α and JNK in cardiomyocytes.

To study whether the TRB3 expression induced by stretch is regulated at the transcriptional level, we cloned the promoter region of rat TRB3 and constructed a luciferase reporter plasmid (pGL3-Luc). The TRB3 promoter construct contains NF-IL6, AARE, ATF-4 and GADD153 binding sites (Fig 3B). As shown in Fig 3C, cyclic stretch for 12 h significantly enhanced TRB3 promoter activity. This result indicates that TRB3 expression is induced at transcriptional level during stretch in cardiomyocytes. Besides, transient transfection of TRB3-mut plasmid and addition of SP600125, TNF-α antibody and etanercept reversed the promoter activity induced by stretch.

### Cyclic stretch induces TRB3 protein expression in cardiomyocytes is through TNF-α

**Fig 4:**
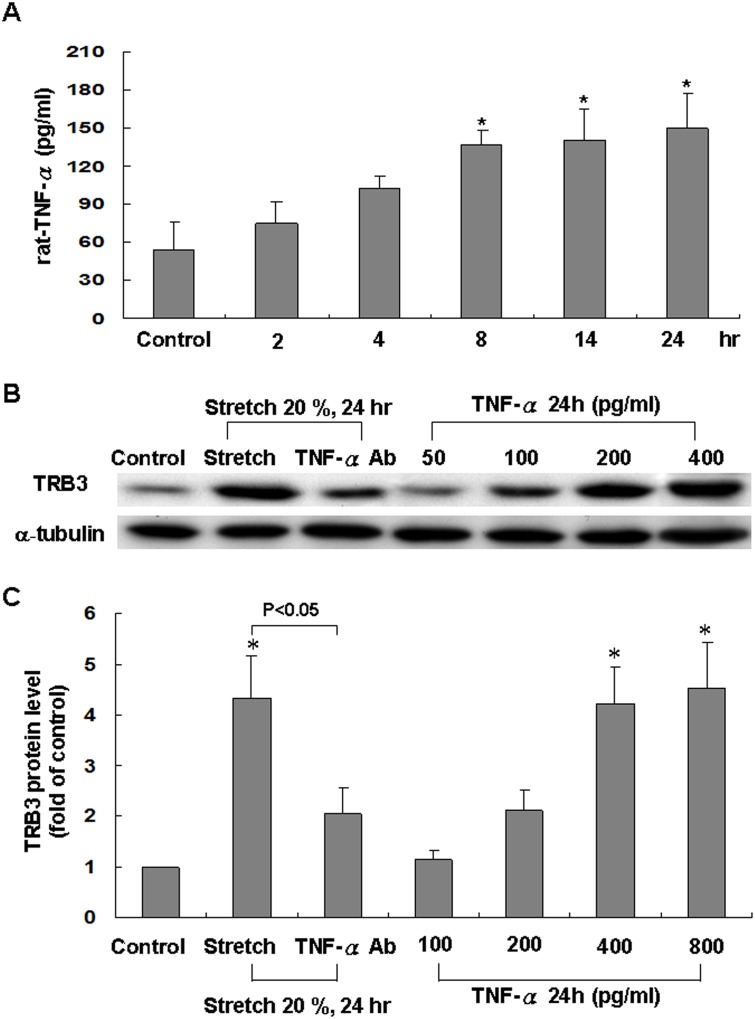
Effects of rat TNF-α on TRB3 in cardiomyocytes. (A) Cyclic stretch enhances release of rat TNF-α from cardiomyocytes subjected to cyclic stretch by 20% for various periods of time (n = 5 per group). *P<0.05 vs. control. (B) Exogenous administration of rat TNF-αincreases TRB3 protein expression. Representative western blots for TRB3 in cardiomyocytes after exogenous administration of rat TNF-α. (C) Quantitative analysis of TRB3 protein levels. The values from treated cardiomyocytes have been normalized to matched α-tubulin measurement and then expressed as a ratio of normalized values to control cells (n = 3 per group). *P<0.05 vs. control.

As shown in Fig 4A, cyclic stretch dramatically increased the TNF-α secretion from cardiomyocytes at 8 h and remained elevated for 24 h. This result indicates that stretch causes secretion of TNF-α from cardiomyocytes.

To explore the direct effect of TNF-α on TRB3 expression in cardiomyocytes, TNF-α at different concentrations was administrated to the cultured medium for 24 h. The effect of TNF-α on TRB3 protein expression was dose-dependent (Fig 4B and 4C). Addition of TNF-α monoclonal antibody 30 min before stretch significantly reversed the expression of TRB3 induced by cyclic stretch. Besides, TRB3 protein expression induced by stretch was not affected by the addition of IFN-γ or IL-6 antibody (S3A and S3B Fig). These findings demonstrate that TNF-α increases TRB3 expression by cyclic stretch in cardiomyocytes.

### Cyclic stretch-induced apoptosis is mediated by TRB3 in cardiomyocytes

**Fig 5:**
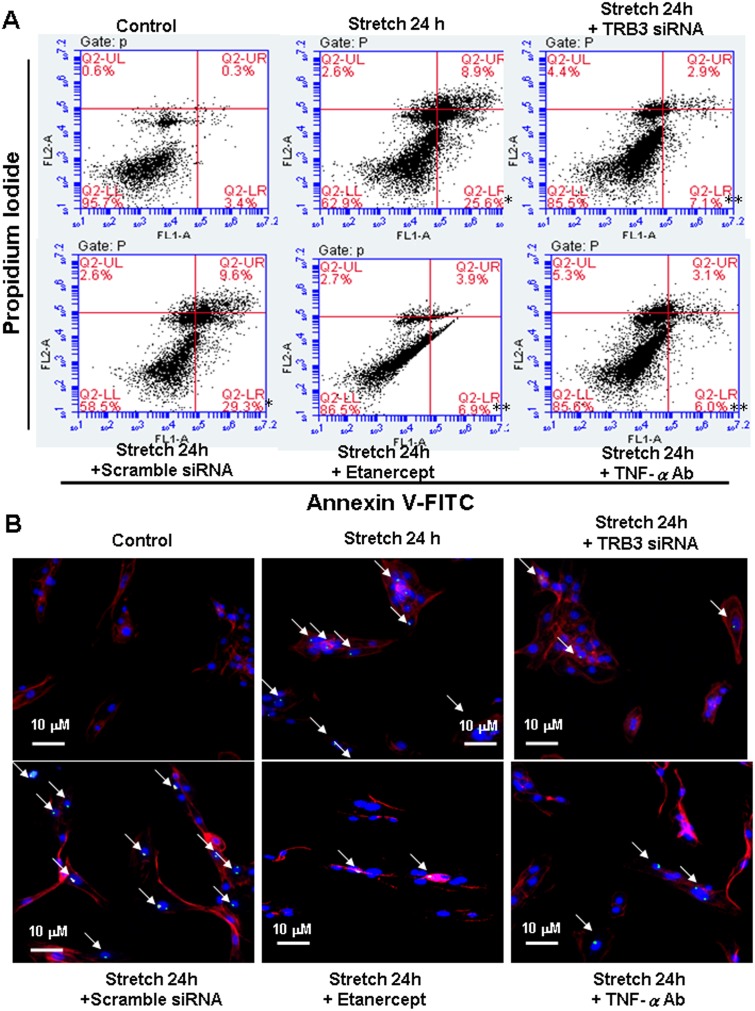
Effect of TRB3 on stretch-induced apoptosis in cardomyocytes. (A) cardiomyocytes were subjected to cyclic stretch 24h, addition of TNF-α Ab, TRB3 siRNA or etanercept (1 μg/ml) before stretch. Quantification of the apoptotic fractions was performed using FACScan. (n = 3). Cells that stain negative for both Annexin V and PI are alive. Cells that stain positive for Annexin V and negative for PI are undergoing apoptosis. Cells that stain positive for both Annexin V and PI are in the end stage of apoptosis called second apoptosis. (n = 3). *P<0.05 vs. control. **P<0.05 vs. stretch 18h. (B) TUNEL assay was made to detect the cardiomyocyte apoptosis under cyclic stretch. Blue color means nucleus stained by DAPI. Red color means cytoskeleton stained by rhodamine. Green color means TUNEL positive nuclei. Representative microscopy images of cardiomyocytes after stretch 24 hr, addition of TNF-α Ab, TRB3 siRNA or etanercept before stretch then stained with by the TUNEL kit. Similar results were observed in another two independent experiments.

Cyclic stretch not only increased the death rate but also decreased the viability of cardiomyocytes measured by a cell counter and MTT assay (S4 Fig). These results suggest that cyclic stretch induces cell death of cardiomyocytes. As shown in Fig 5A, apoptosis was assessed by PI/annexin V double staining and FACS analysis. The percentage of cells stained with annexin V was elevated following stretch for 24 h. These increases of annexin V positive cells induced by stretch were significantly reversed by TNF-α antibody, TRB3 siRNA and etanercept. The TUNEL assay was also used to confirm the presence of apoptotic nuclei after cyclic stretch (Fig 5B and S5A Fig). A significant increase in TUNEL-positive nuclei was present after stretch for 24 h. These increases of TUNEL-positive nuclei in cardiomyocytes enhanced by stretch were significantly reversed by TNF-α antibody, TRB3 siRNA and etanercept. TRB3 protein expression enhanced by stretch was inhibited by the addition of TRB3 siRNA (S3C and S3D Fig). These findings indicated that TRB3 mediates stretch-induced cardiomyocyte apoptosis. We further investigate the downstream regulation between TRB3 and apoptosis. As shown in S5B and S5C Fig, cyclic stretch reduced the phospho Akt protein expression. Addition of TRB3 siRNA 24 h prior to stretch reversed the decline of phospho Akt. This data may indicate that TRB3 mediating cardiomyocyte apoptosis under stretch is through inhibiting Akt pathway.

### In vivo AV shunt enhances myocardial TRB3 protein and mRNA expression

**Fig 6:**
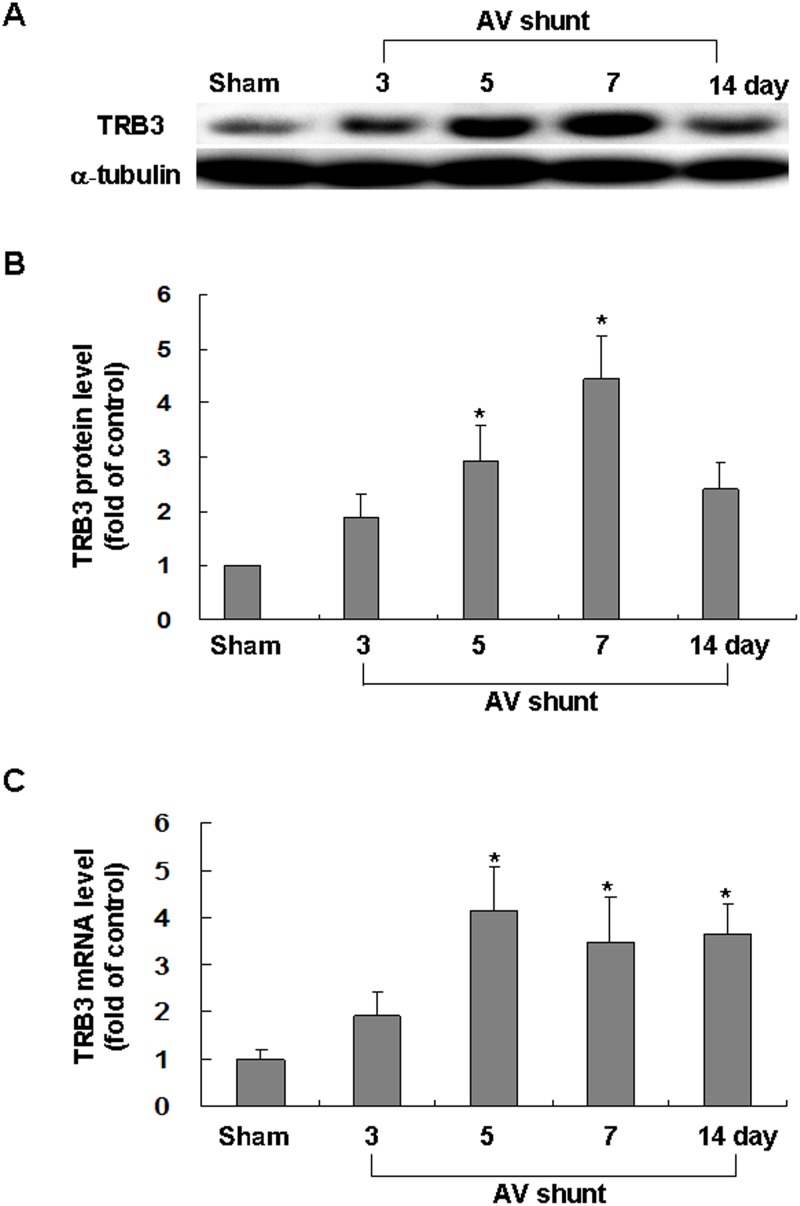
Effect of *in vivo* model of aorta-caval shunt (AV shunt) on myocardial TRB3 protein levels. (A) Representative Western blots for TRB3 in rat myocardium after short-term induction of AV shunt. (B) Quantitative analysis of TRB3 protein levels. The values have been normalized to α-tubulin measurement and then expressed as a ratio of normalized values to TRB3 protein in sham. (n = 3 per group). **P<0.05 vs. control. (C) Fold increases in TRB3 mRNA as a result of induction of AV shunt. The values from experiment groups have been normalized to match GADPH measurement and then expressed as a ratio of normalized values to mRNA in sham group. *P<0.05 vs. sham group. (n = 3 per group).

AV shunt was performed to investigate whether TRB3 expression was increased under volume-overload in vivo. The TRB3 protein expression in rat myocardium significantly increased at 5 and 7 days after induction of AV shunt (Fig 6A and 6B). Real-time PCR also showed that TRB3 mRNA was up-regulated after AV shunt (Fig 6C). These findings indicate TRB3 was enhanced by volume overload in rat myocardium.

### Etanercept inhibits TRB3 protein expression and myocardial apoptosis induced by AV shunt

**Fig 7:**
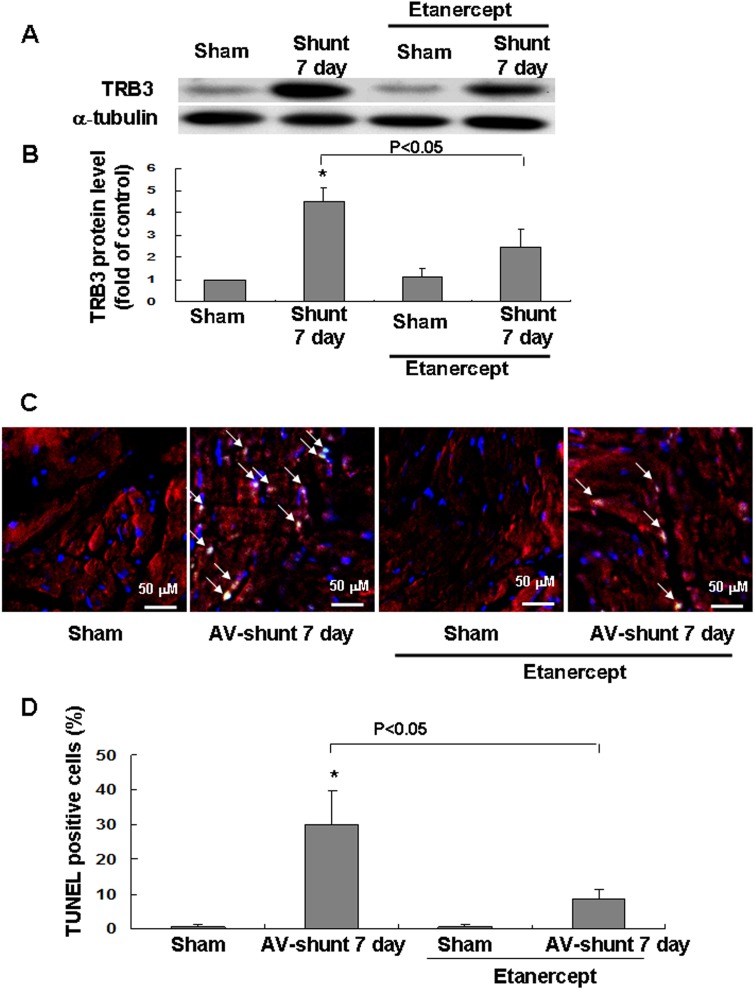
Effect of AV-shunt and treatment with etanercept (1 mg/kg, purchased from BOC Science, NY, USA) on TRB3 protein expression and cardiac myocytes apoptosis. (A) Representative Western blots analysis for TRB3 after induction of AV shunt with or without treatment with etanercept. (B) Quantitative analysis of TRB3 protein levels. The values from experimental groups have been normalized to values in sham group. (n = 5 per group). *P < 0.05 vs. sham group. (n = 5 per group). (C) TUNEL staining of cardiac myocytes after induction of AV shunt with or without treatment with etanercept. Blue color means nucleus stained by DAPI. Red color means desmin. Green color means TUNEL positive nuclei. (D) Quantitative analysis of TUNEL positive cells. *P < 0.05 vs. sham group. (n = 3 per group).

As shown in Fig 7A and 7B, treatment with etanercept significantly reversed the increase of TRB3 protein induced by AV shunt. However, treatment with etanercept in the sham group did not affect the protein expression of TRB3. Besides, AV shunt significantly enhanced apoptotic nuclei. Treatment with etanercept significantly reduced TUNEL positive cells (Fig 7C and 7D) and caspase 3 expression (S6 Fig) induced by AV shunt. These findings demonstrate that etanercept inhibits myocardial TRB3 protein expression and apoptosis induced by AV shunt.

## Discussion

In this study, we demonstrated several significant findings. (1) Cyclic stretch induces TRB3 expression in rat cardiomyocytes; (2) cyclic stretch upregulates TNF-α expression in cardiomyocytes; (3) JNK MAP kinase and GADD153 transcription factor are involved in the signaling pathway of TRB3 induction; (4) apoptosis of cardiomyocyte induced by cyclic stretch is TRB3 dependent; (5) In vivo acute hemodynamic overload enhances rat myocardium TRB3 expression; (6) Etanercept inhibits myocardial TRB3 expression and apoptosis induced by AV shunt. TRB3 was increased in both a time- and load- dependent manner by cyclic stretch. Cyclic stretch of cardiomyocytes upregulated both TRB3 protein and mRNA expression.

Our study demonstrated that stretch induced TNF-α secretion and TNF-α mediated cyclic stretch-induced expression of TRB3 in cardiomyocytes. Boltzen et al. have demonstrated that tissue factor and full-length tissue factor protect cardiomyocytes against TNF-α-induced apoptosis [23]. Besides, Lv et al. also indicated that berberine could reduce norepinephrine-induced apoptosis in neonatal rat cardiomyocytes through inhibiting the ROS-TNF-α-caspase signaling pathway [24]. These results are consistent with our data that TNF-α plays an important role in cardiomyocyte apoptosis. However, TNF-α may have an opposite function in other cardiac vascular cells like vascular smooth muscle cells (VSMCs). Chen et al. have demonstrated that naringenin inhibiting TNF-α induced VSMCs proliferation is through heme oxygenase-1 [25]. In general, the character of TNF-α in cardiomyocytes is injury rather than protection.

We demonstrated that cyclic stretch induced TRB3 protein expression and GADD153-DNA binding activity is regulated by JNK pathway since they are both inhibited by JNK inhibitor or siRNA. Kristiansen et al. have suggested that TRB3 is a potential target of the mixed lineage kinase-JNK-c-Jun pathway in sympathetic neurons cultured in the absence of nerve growth factor (NGF) [26]. However, Formoso et al. have demonstrated that TRB3 R4 variant is associated with increased MAPK signaling in human umbilical vein endothelial cells [27]. Kiss-Toth et al. also indicated that tribbles control MAPK cascades including ERK, p38 and JNK pathway [28]. These results suggest that there is a feedback relationship between TRB3 and JNK. Our results indicate that the JNK MAP kinase pathway is involved in the induction of TRB3 by stretch and mediates the increased binding activity of GADD153.

Our data indicate that TRB3 expression induced by cyclic stretch is through GADD153 pathway. Previously, we demonstrated that cyclic stretch could enhance GADD153 expression [19]. In this study, we demonstrate that TRB3 protein expression induced by stretch is reversed by GADD153 siRNA. Wali et al. also demonstrated that knockdown of GADD153 by specific siRNAs attenuated gamma-tocotrienol-induced TRB3 expression [29]. GADD153, a stress inducible gene, is involved in ER stress-mediated apoptosis [30]. GADD153 is one of the C/EBP family protein that forming heterodimers with other C/EBP proteins such as ATF-4 to inhibit the DNA binding activity to classical C/EBP target gene and their transcriptional activity [31]. GADD153-C/EBP heterodimer could bind to GADD153-C/EBP specific binding site and act as a transcription factor [32]. We demonstrate that TRB3 promoter activity regulated by GADD153 under stretch is at transcriptional level. Bromati et al. demonstrated that GADD153 and ATF4 cooperated to activate TRB3 promoter activity [33]. Wennemers et al. indicated that induction of TRB3 by hypoxia was regulated via the PERK/ATF4/GADD153 pathway of the unfolded protein response [34]. These results reveal that GADD153 plays a crucial role in TRB3 transcription activity.

In this study, our data revealed that cardiomyocyte apoptosis induced by stretch is mediated by TRB3. Honsho et al. demonstrated that IL-1β deficient mice markedly increased caspase-3 activities and cardiomyocyte apoptosis induced by cyclic stretch [35]. However, De Jong et al. reported that a 24 hr 15% stretch resulted induction of the fetal gene program and cell death but no apoptosis was observed [36]. Therefore, it is possible that part of the TUNEL staining may represent other forms of cell death due to stresses such as hypoxia. In this study, the intensity we used to induce cardiomyocyte apoptosis is 20%. The different strength of stretch may explain the discrepancy. Avery et al. reported that transgenic TRB3 mice were sensitized to cardiac myocyte apoptosis in the infarct border zone after myocardial infarction [20]. Besides, Zhou et al. have demonstrated that emodin induces BV-2 cell apoptosis through TRB3 [37]. These results are consistent with our finding that TRB3-mediated apoptosis. Our data found that the reduction of phospho Akt protein expression induced by cyclic stretch is reversed by TRB3 siRNA. TRB3 has been indicated to inhibit the activity of Akt protein kinases [20]. Raaz et al. also demonstrated that 24 h stretch-induced reduction of the anti-apoptotic messenger pAkt in human pulmonary microvascular endothelial cell [38].

Hemodynamic overload includes pressure overload and volume overload. Transthoracic aortic banding is a pressure overload to induce cardiac hypertrophy. AV shunt is a volume overload to induce cardiac dilatation. Cardiomyocytes are more stretched in the dilated left ventricle than in the hypertrophic left ventricle. Therefore, we used AV shunt as an in vivo study. We provided the first evidence that the increased myocardial TRB3 expression in acute haemodynamic overload as in aorta-caval shunt. Averyet al. indicated that TRB3 expression was sensitized to infarct expansion in transgenic TRB3 mice after myocardial infarction [20]. Koh et al. have reported that high-fat feeding in mice and type 2 diabetes in humans dramatically enhances TRB3 in skeletal muscle [39]. Besides, other study also demonstrated that TRB3 expression was upregulated in transgenic OVE26 and Akt2-KO diabetic mouse models [40]. These results suggest that TRB3 expression could be increased under abnormal stress in animal model and humans. We found that etanercept could inhibit the cardiomyosytes TRB3 expression and apoptosis under AV-shunt. Gutkowska et al. reported that etanercept could prevent cardiomyocyte apoptosis induced by reduction of uteroplacental perfusion pressure in pregnant rats [41]. These findings implicate that TRB3 could be a therapeutic candidate in cardiovascular disease.

**Fig 8:**
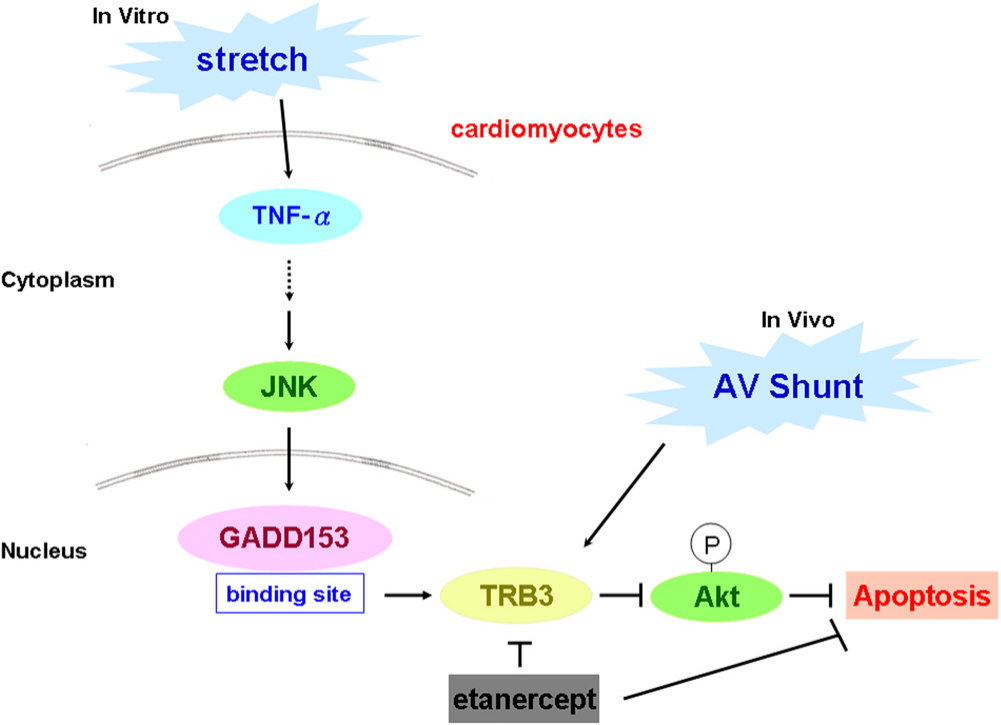
Schematic diagram of signal pathway responsible for TRB3 mediated apoptosis of cardiomyocyte under mechanical stretch. The TRB3 expression induced by stretch is through TNF-α, JNK and GADD153 pathway. Eanercept inhibits TRB3 and thus attenuates the apoptosis of cardiomyocyte induced by stretch.

In summary, we demonstrate for the first time that cyclic stretch induces TRB3 expression in cultured rat cardiomyocytes. The stretch-induced TRB3 is regulated by TNF-α, JNK MAP kinase and GADD153 pathway. Apoptosis of cardiomyocytes induced by stretch is TRB3 dependent. Our result also reveals that etanercept inhibits myocardium TRB3 expression and apoptosis induced by AV shunt. Fig 8 summaries the molecular mechanism and signaling pathway of cardiomyocyte apoptosis induced by mechanical stretch and AV shunt. Further studies can help to clarify the specific role of TRB3 in cardiomyocyte apoptosis and heart failure.

## Conclusions

Apoptosis of cardiomyocytes induced by stretch is TRB3 dependent and Etanercept, a specific TNF-α inhibitor, could inhibit TRB3 expression and apoptosis induced by mechanical stress in cardiomyocytes.

## Supporting Information

S1 Fig Effects of JNK siRNA on phospho and total JNK protein expression induced by cyclic stretch in cardiomyocytes. (A) Representative Western Blots for phosphor or total JNK protein levels in cardiomyocytes subjected to cyclic stretch in the absence of JNK siRNA. (B) Quantitative analysis of TRB3 protein levels. The values from stretched cardiomyocytes have been normalized to matched α-tubulin measurement and then expressed as a ratio of normalized values to protein in control group (n = 3 per group). *P < 0.05 vs. phospho JNK control. **P < 0.05 vs. total JNK control.
(TIF)

S2 Fig Effects of GADD153 siRNA on TRB3 and GADD153 protein expression induced by cyclic stretch in cardiomyocytes. (A) Representative Western Blots for TRB3 protein levels in cardiomyocytes subjected to cyclic stretch in the absence of GADD153 siRNA. (B) Quantitative analysis of TRB3 protein levels. The values from stretched cardiomyocytes have been normalized to matched α-tubulin measurement and then expressed as a ratio of normalized values to protein in control group (n = 3 per group). *P < 0.05 vs. control. (C) Representative Western Blots for GADD153 protein levels in cardiomyocytes subjected to cyclic stretch in the absence of GADD153 siRNA. (D) Quantitative analysis of GADD153 protein levels. The values from stretched cardiomyocytes have been normalized to matched α-tubulin measurement and then expressed as a ratio of normalized values to protein in control group (n = 3 per group). *P < 0.05 vs. control.
(TIF)

S3 Fig Effects of various cytokine antibody and TRB3 siRNA on TRB3 protein expression induced by cyclic stretch in cardiomyocytes. (A) Representative Western Blots for TRB3 protein levels in cardiomyocytes subjected to cyclic stretch in the absence of IFN-γ, TNF-α and IL-6 Ab. (B) Quantitative analysis of TRB3 protein levels. The values from stretched cardiomyocytes have been normalized to matched α-tubulin measurement and then expressed as a ratio of normalized values to protein in control group (n = 3 per group). *P < 0.05 vs. control. (C) Representative Western Blots for TRB3 protein levels in cardiomyocytes subjected to cyclic stretch in the absence of TRB3 or scramble siRNA. (D) Quantitative analysis of TRB3 protein levels. The values from stretched cardiomyocytes have been normalized to matched α-tubulin measurement and then expressed as a ratio of normalized values to protein in control group (n = 3 per group). *P < 0.05 vs. control.
(TIF)

S4 Fig Effect of TRB3 on stretch-induced survival and death rate in cardiomyocytes. Quantitative analysis of trypan blue exclusion (A) and MTT assay (B) for cardiomyocytes viability after stretch and addition of etanercept, TRB3 or JNK siRNA. (n = 5). *P<0.05 vs. control.
(TIF)

S5 Fig Effects of TRB3 siRNA on cardiomyocyte apoptosis and phospho Akt protein expression induced by cyclic stretch in cardiomyocytes. (A) Quantitative analysis of TUNEL positive cardiomyocytes were subjected to cyclic stretch 24h, addition of TNF-α Ab, TRB3 siRNA or etanercept (1 μg/ml) before stretch. *P < 0.05 vs. control group. (n = 4 per group). (B) Representative Western Blots for phosphor Akt protein levels in cardiomyocytes subjected to cyclic stretch in the absence of TRB3 siRNA. (C) Quantitative analysis of phospho Akt protein levels. The values from stretched cardiomyocytes have been normalized to matched total Akt measurement and then expressed as a ratio of normalized values to protein in control group (n = 3 per group). *P < 0.05 vs. control.
(TIF)

S6 Fig Effect of AV-shunt and treatment with etanercept (1 mg/kg) on caspase 3 protein expression. Blue color means nucleus stained by DAPI. Red color means desmin. Green color means caspase 3. Similar results were observed in another two independent experiments.
(TIF)

S1 Methods The supplementary methods include detail description of real-time quantitative PCR, western blot analysis, electrophoretic mobility shift assay (EMSA), construction of small interfering RNA (siRNA), cytotoxicity study, terminal deoxynucleotidyl transferase-mediated dUTP nick-end labeling (TUNEL) assay and immunohistochemistry. (DOC)
