# Supplementary figures and images for: Mechanical Stretch Induces Apoptosis Regulator TRB3 in Cultured Cardiomyocytes and Volume-Overloaded Heart
Source: PLoS One. 2015 Apr 21;10(4):e0123235. doi: 10.1371/journal.pone.0123235 (PMC4405267; doi:10.1371/journal.pone.0123235)

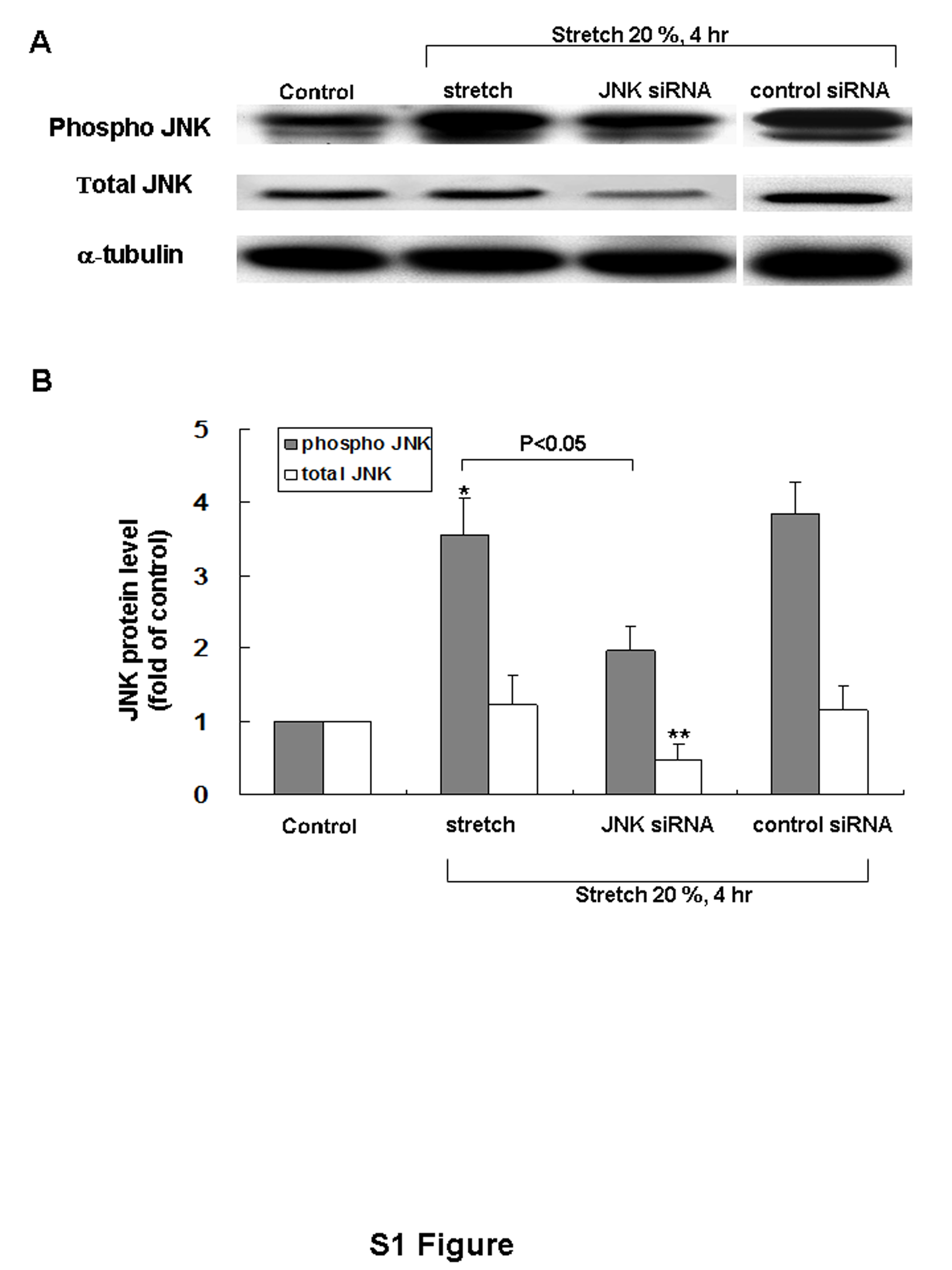

Supplement: S1 Fig — (A) Representative Western Blots for phosphor or total JNK protein levels in cardiomyocytes subjected to cyclic stretch in the absence of JNK siRNA. (B) Quantitative analysis of TRB3 protein levels. The values from stretched cardiomyocytes have been normalized to matched α-tubulin measurement and then expressed as a ratio of normalized values to protein in control group (n = 3 per group). *P < 0.05 vs. phospho JNK control. **P < 0.05 vs. total JNK control. (TIF) [file pone.0123235.s001.tif]

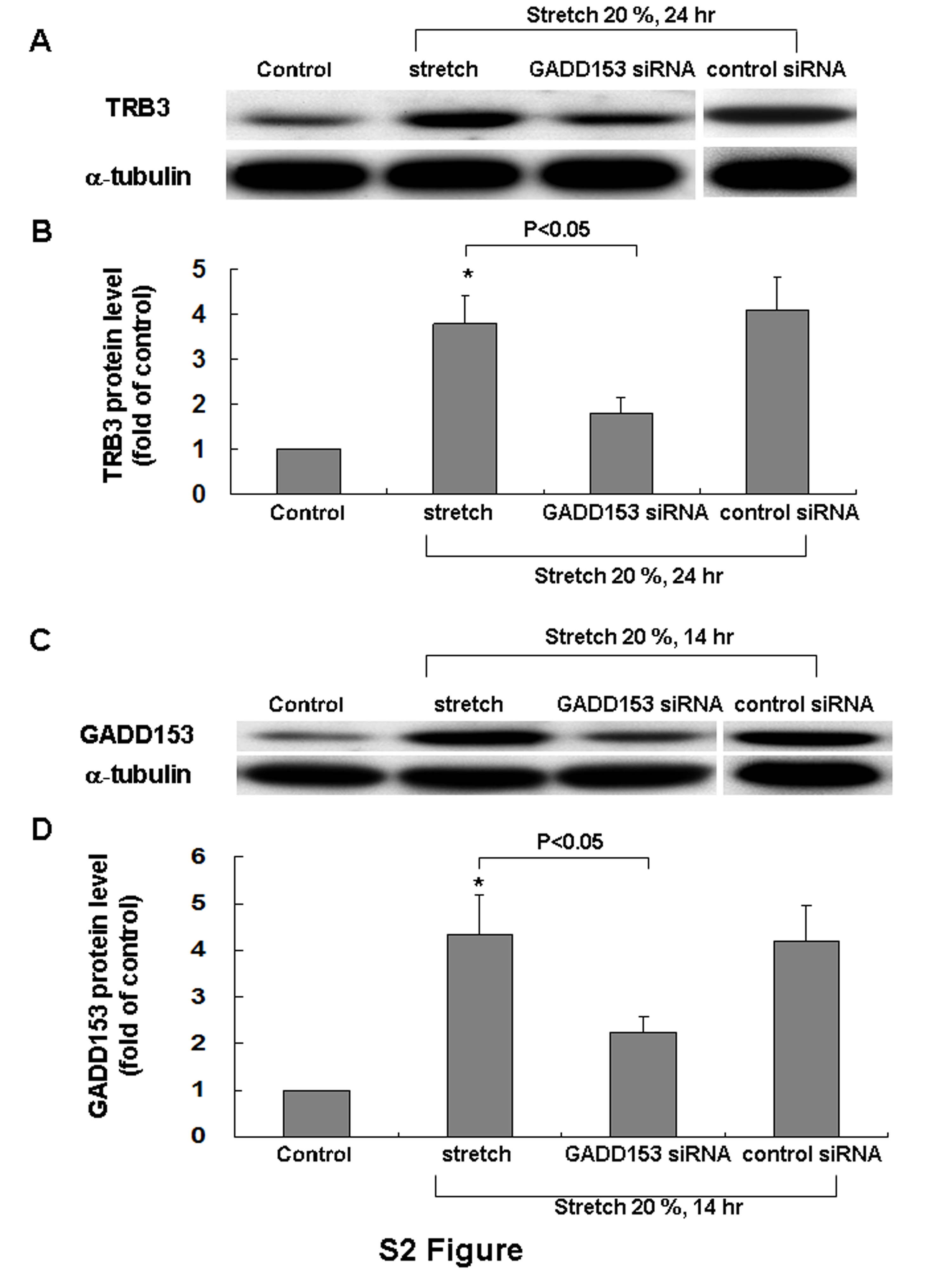

Supplement: S2 Fig — (A) Representative Western Blots for TRB3 protein levels in cardiomyocytes subjected to cyclic stretch in the absence of GADD153 siRNA. (B) Quantitative analysis of TRB3 protein levels. The values from stretched cardiomyocytes have been normalized to matched α-tubulin measurement and then expressed as a ratio of normalized values to protein in control group (n = 3 per group). *P < 0.05 vs. control. (C) Representative Western Blots for GADD153 protein levels in cardiomyocytes subjected to cyclic stretch in the absence of GADD153 siRNA. (D) Quantitative analysis of GADD153 protein levels. The values from stretched cardiomyocytes have been normalized to matched α-tubulin measurement and then expressed as a ratio of normalized values to protein in control group (n = 3 per group). *P < 0.05 vs. control. (TIF) [file pone.0123235.s002.tif]

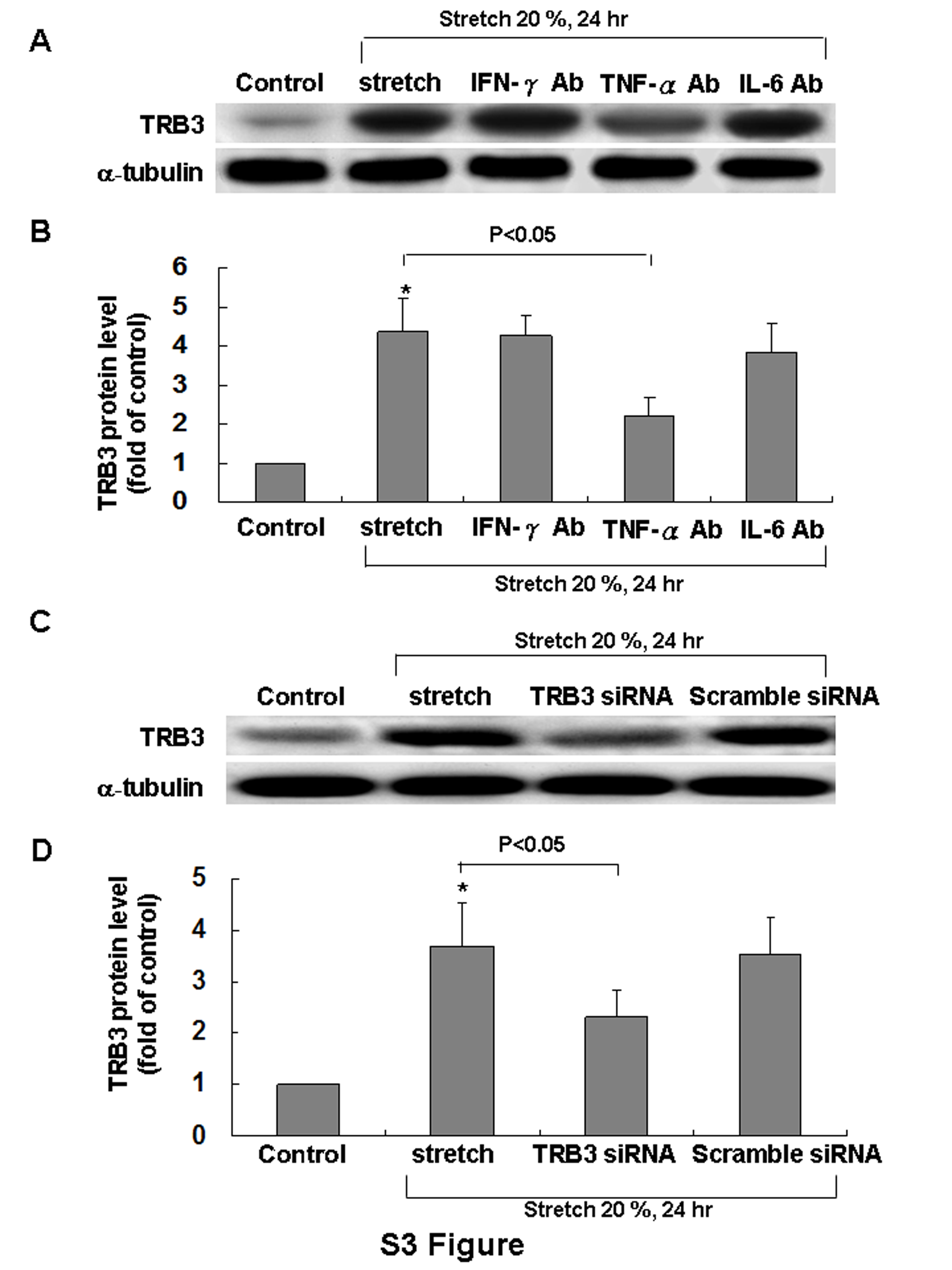

Supplement: S3 Fig — (A) Representative Western Blots for TRB3 protein levels in cardiomyocytes subjected to cyclic stretch in the absence of IFN-γ, TNF-α and IL-6 Ab. (B) Quantitative analysis of TRB3 protein levels. The values from stretched cardiomyocytes have been normalized to matched α-tubulin measurement and then expressed as a ratio of normalized values to protein in control group (n = 3 per group). *P < 0.05 vs. control. (C) Representative Western Blots for TRB3 protein levels in cardiomyocytes subjected to cyclic stretch in the absence of TRB3 or scramble siRNA. (D) Quantitative analysis of TRB3 protein levels. The values from stretched cardiomyocytes have been normalized to matched α-tubulin measurement and then expressed as a ratio of normalized values to protein in control group (n = 3 per group). *P < 0.05 vs. control. (TIF) [file pone.0123235.s003.tif]

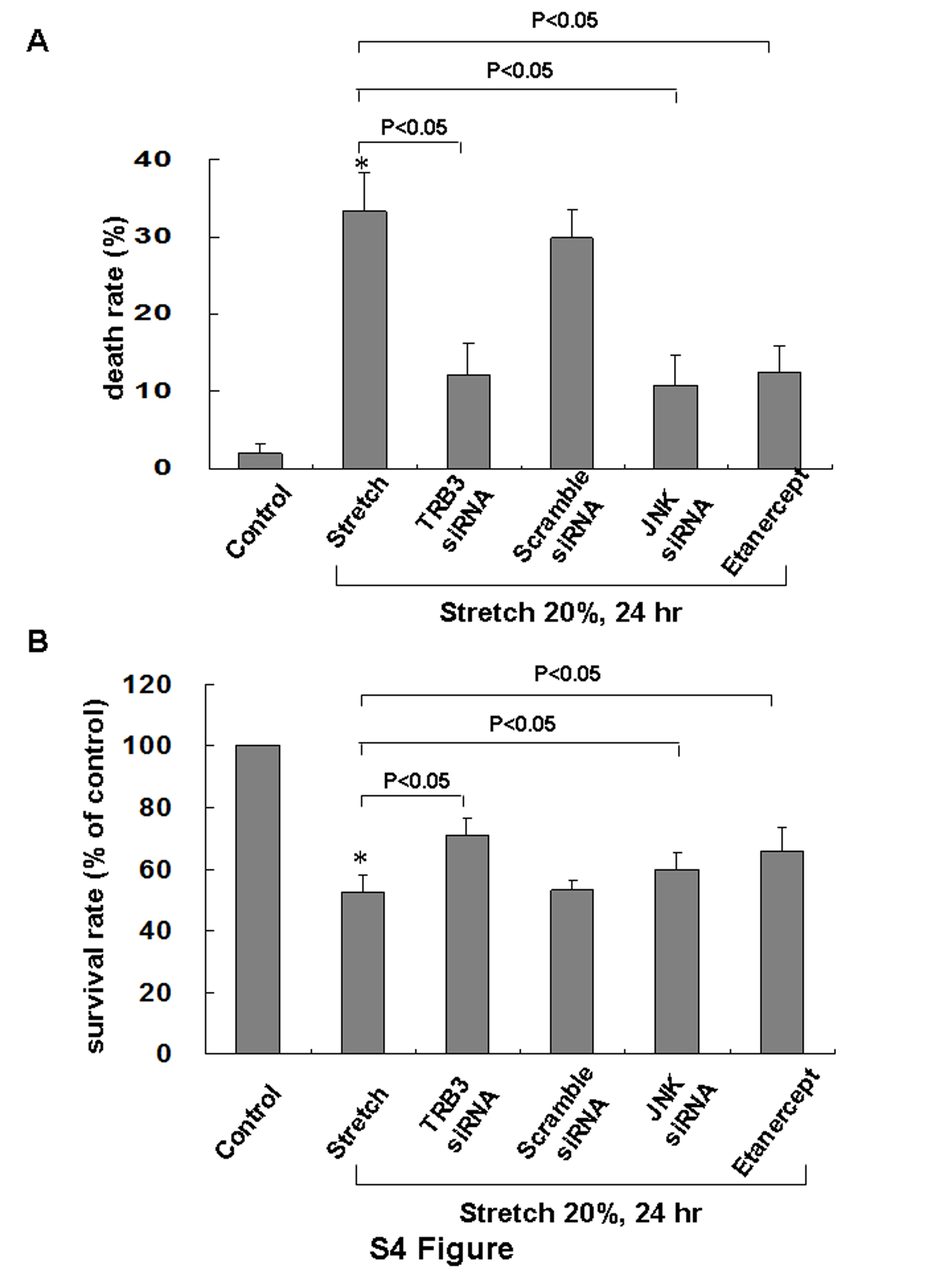

Supplement: S4 Fig — Quantitative analysis of trypan blue exclusion (A) and MTT assay (B) for cardiomyocytes viability after stretch and addition of etanercept, TRB3 or JNK siRNA. (n = 5). *P<0.05 vs. control. (TIF) [file pone.0123235.s004.tif]

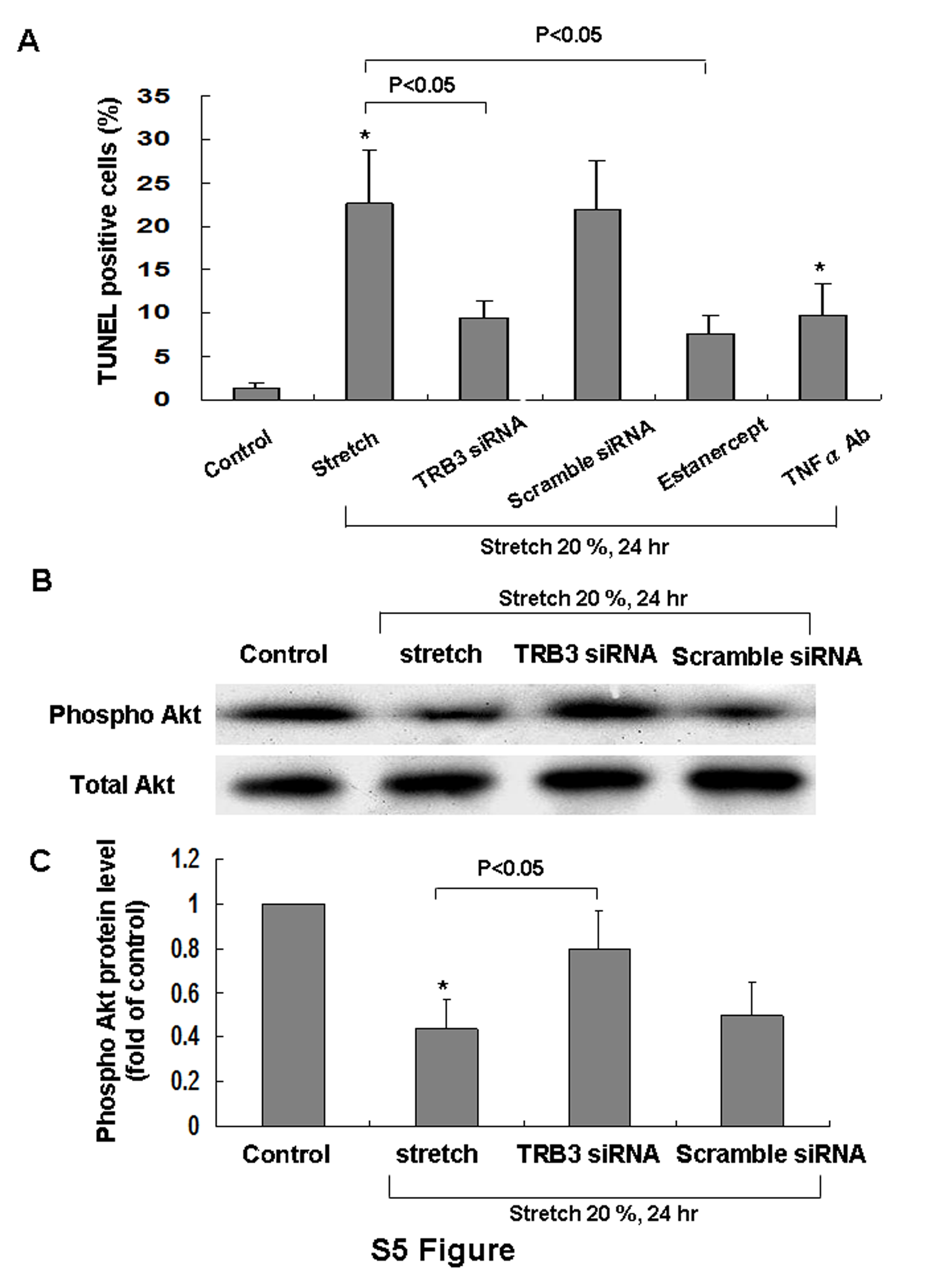

Supplement: S5 Fig — (A) Quantitative analysis of TUNEL positive cardiomyocytes were subjected to cyclic stretch 24h, addition of TNF-α Ab, TRB3 siRNA or etanercept (1 μg/ml) before stretch. *P < 0.05 vs. control group. (n = 4 per group). (B) Representative Western Blots for phosphor Akt protein levels in cardiomyocytes subjected to cyclic stretch in the absence of TRB3 siRNA. (C) Quantitative analysis of phospho Akt protein levels. The values from stretched cardiomyocytes have been normalized to matched total Akt measurement and then expressed as a ratio of normalized values to protein in control group (n = 3 per group). *P < 0.05 vs. control. (TIF) [file pone.0123235.s005.tif]

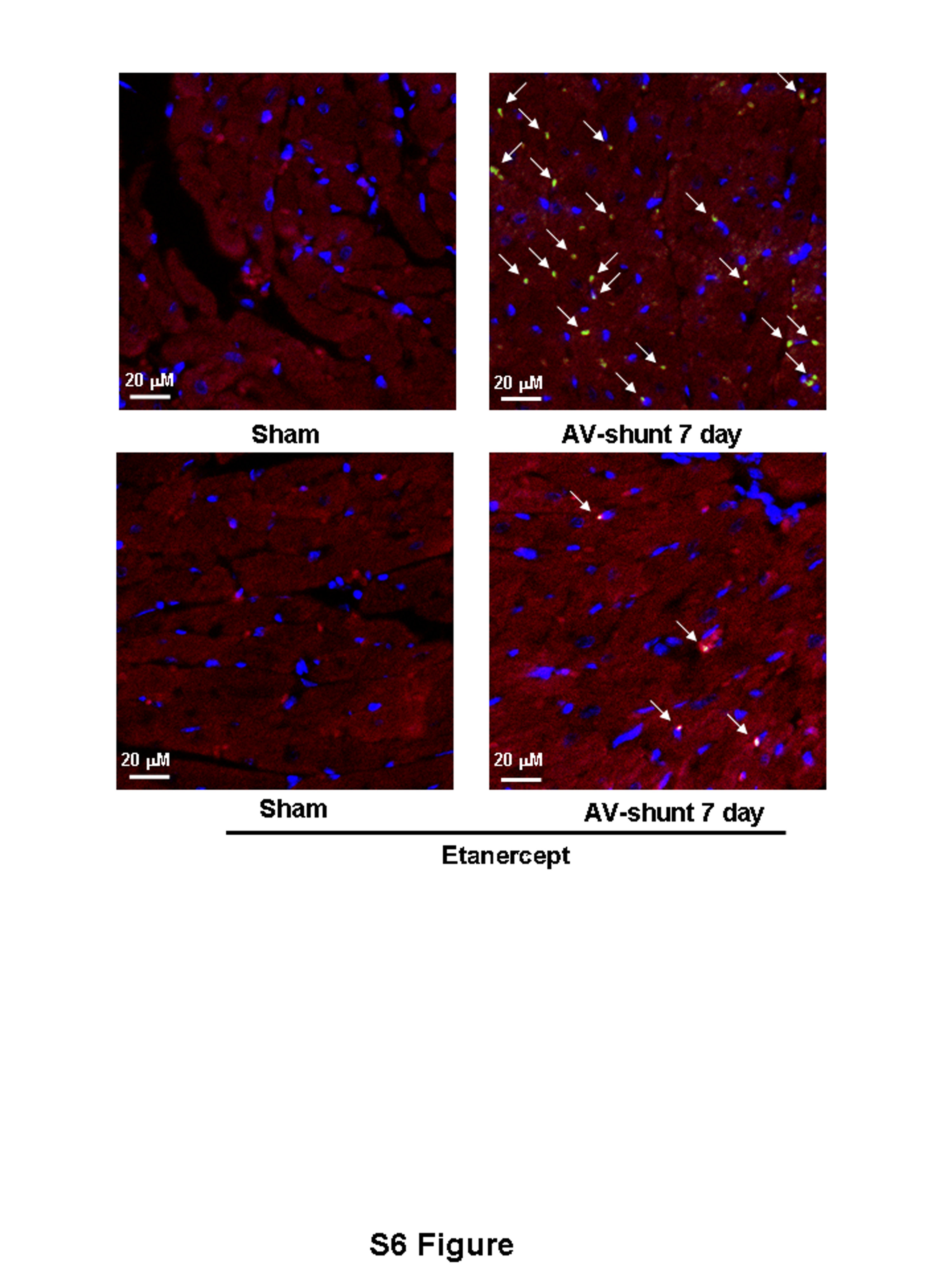

Supplement: S6 Fig — Blue color means nucleus stained by DAPI. Red color means desmin. Green color means caspase 3. Similar results were observed in another two independent experiments. (TIF) [file pone.0123235.s006.tif]
