# Supplementary material for: Mechanical Stretch Induces Apoptosis Regulator TRB3 in Cultured Cardiomyocytes and Volume-Overloaded Heart
Source: PLoS One. 2015 Apr 21;10(4):e0123235. doi: 10.1371/journal.pone.0123235 (PMC4405267; doi:10.1371/journal.pone.0123235)
Supplement: S1 Methods — (DOC) [file pone.0123235.s007.doc]

**Supplementary Methods**

**Real-time Quantitative PCR**

A Lightcycler (Roche Diagnostics, Mannheim, Germany) was used for real-time PCR. cDNA was diluted 1 in 10 with nuclease-free water. 2 μL of the solution was used for the Lightcycler SYBR–Green mastermix (Roche Diagnostics): 0.5 μmol/L primer, 5 mmol/L magnesium chloride, and 2 μL Master SYBR-Green in nuclease-free water in a final volume of 20 μL. The primer used for TRB3, 5’-d(TCGCTGACCGTGAGAGGAA)-3’ (forward) and 5’-d(GCAAGATGAGCCCGTGCTT)-3’ (reverse). GAPDH, 5’-d(CATCACCATCTTCCAGG AGC) (forward) and 5’-d(GGATGATGTTCTGGGCTGCC)-3’ (reverse). The initial denaturation phase for rat TRB3 was 5 min at 95 ℃ followed by an amplification phase as detailed below: denaturation at 95 ℃ for 10sec; annealing at 63℃ for 7sec; elongation at 72 ℃ for 8sec; detection at 79 ℃ and for 45cycles. Amplification, fluorescence detection, and post-processing calculation were performed using the Lightcycler apparatus. Individual PCR products were analyzed for DNA sequence to confirm the purity of the product.

**Western blot analysis**

Cardiomyocytes or myocardium were homogenized in modified RIPA buffer (50 mmol/L tris [pH 7.4], 1% IGEPAL CA-630 (Sigma), 0.25% sodium deoxycholate, 150 mmol/L NaCl, 1 m mol/L EDTA, 1 mmol/L phenylmethylsulfonyl fluoride, and 1 μg/mL aprotinin, leupetin, and pepstatin). Equal amounts of protein (80μg) were loaded into a 12.5% SDS–polyacrylamide minigel, followed by electrophoresis. Protein samples were mixed with sample buffer, boiled for 10 min, separated by SDS–PAGE under denaturing conditions, and electroblotted to PVDF membranes. The blots were incubated overnight in PBS containing 0.1% Tween 20 (TPBS) which containing 5% milk to block nonspecific binding of the antibody. Then membranes were incubated with anti-TRB3 (1:200; Santa Cruz Biotechnology Inc., Santa Cruz, CA, USA), washed, and incubated with horseradish peroxidase-conjugated secondary antibody. Signals were visualized by chemiluminescent detection. Equal protein loading of the samples was further verified by staining monoclonal antibody α-tubulin. All Western blots were quantified using densitometry. Anti-phospho JNK (1:200; Santa Cruz Biotechnology Inc., Santa Cruz, CA, USA); Anti-total JNK (1:200; Santa Cruz Biotechnology Inc., Santa Cruz, CA, USA); Anti-GADD153 (1:200; Santa Cruz Biotechnology Inc., Santa Cruz, CA, USA); Anti-phospho Akt (1:1000; Cell signaling Technology, Danvers, MA, USA); Anti-total Akt (1:1000; Cell signaling Technology, Danvers, MA, USA).

**EMSA**

Nuclear protein concentrations from cultured cardiomyocytes were determined by Biorad protein assay. Consensus and control oligonucleotides (Santa Cruz Biotechnology Inc., Santa Cruz, CA, USA) were labeled by polynucleotides kinase incorporation of [γ^32^-P]ATP’. After the GADD153 binding site: TGGTGCAATCCCC was radiolabeled [22], the nuclear extracts (4 μg of protein in 2 μl of nuclear extract) were mixed with 20 pmol of the appropriate [γ^32^-P]ATP-labeled consensus or mutant oligonucleotide in a total volume 20 μl for 30 min at room temperature. The samples were then resolved on a 4％ polyacrylamide gel. Gels were dried and imaged by autoradiography. Controls were performed in each case with mutant oligonucleotides or cold oligonucleotides to compete with labeled sequence.

**Construction of small interfering RNA (siRNA)**

Cardiomyocytes were transfected with TRB3 annealed siRNA oligonucleotide (Dharmacon Inc., Lafayette, CO, USA) to knockdown gene expression. The siRNA was transfected into cardiomyocytes using a low pressure-accelerated gene gun (Bioware Technologies, Taipei, Taiwan) essentially following the protocol from the manufacturer. In brief, 800 ng of siRNA was suspended in 5 ml of PBS and was delivered to the cardiomyocytes at a helium pressure of 776 mmHg. The transfective efficiency of using this method is 30%. After overnight incubation, cells were stretched and subjected to analysis by western blot, EMSA, immunohistochemistry and detection of apoptosis.

**Cytotoxicity study**

Cardiomyocytes were adjusted to 3 × 10^4^ cells/ml in F – 10 culture medium. After stretch for 24 h, the medium was aspirated off and 0.5 mg/ml MTT solution was added and incubation continued for another 4 h. At the end of the incubation period, the MTT solution was removed from the attached cells and the converted dye crystals were dissolved with DMSO. Absorbency of converted dye was measured at a wavelength of 570 nm. For detection of cell injury induced by stretch, cell viability after application of stretch was monitored by trypan blue staining.

**Terminal deoxynucleotidyl transferase-mediated dUTP nick-end labeling (TUNEL) assay**

DNA fragmentation was determined by TUNEL using the ApopTag peroxidase in situ apoptosis detection kit (Chemicon International, Temecula, CA). At the end of stretch, cardiomyocytes were fixed in 4% paraformaldehyde for 10 min followed by a staining procedure according to manufacturer’s protocol. In this method, DNA strand breaks were detected by enzymatically labeling the free 3’-OH termini with modified nucleotides with terminal deoxynucleotidyl transferase (TdT). DNA fragments were then labeld with digoxigenin and bound to anti-digoxigenin antibody conjugated to horseradish peroxidase. The peroxidase conjugate produces a localized stain which can be detected.

**Immunohistochemistry**

The Left ventricular tissue was harvested and fixed in 10% formaldehyde and sliced into 5-*μ*m-thick paraffin sections. For immunohistochemical stain, the slides were postfixed in 4% paraformaldehyde for 20 min, treated in 3% hydrogen peroxide/PBS for 25 min, blocked in 5% normal rabbit serum for 20 min, blocked with biotin/ avidin for 15 min each, and incubated with rabbit anti-caspase 3 (Santa Cruz Biotechnology, Santa Cruz) and goat anti-desmin (Santa Cruz Biotechnology, Santa Cruz) at 1:500 for overnight at 4℃, then incubated with donkey-anti rabbit (FITC) IgG and donkey-anti goat (TRITC) IgG (Immuno Research) at 1:500 for 40 min, and Vector Elite ABC biotin-avidin-peroxidase complex for 30 min; Fluorescence signals were obtained with a confocal microscope (Nikon D-ECLIPSE) and assayed using its associated image processing and analysis software.
